# Frequency of Early Intervention Sessions and Vocabulary Skills in Children with Hearing Loss

**DOI:** 10.3390/jcm10215025

**Published:** 2021-10-28

**Authors:** Mallene Wiggin, Allison L. Sedey, Christine Yoshinaga-Itano, Craig A. Mason, Marcus Gaffney, Winnie Chung

**Affiliations:** 1 Department of Speech, Language, and Hearing Sciences, University of Colorado-Boulder, 409 UCB, Boulder, CO 80309, USA; allison.sedey@colorado.edu; 2 Colorado School for the Deaf and the Blind, 33 N. Institute St., Colorado Springs, CO 80903, USA; 3 Institute of Cognitive Science, University of Colorado-Boulder, 594 UCB, Boulder, CO 80309, USA; christie.yoshi@colorado.edu; 4 Centre for Deaf Studies, University of Witwatersrand, Johannesburg 2050, South Africa; 5 School of Learning and Teaching, University of Maine, Orono, ME 04469, USA; craig.mason@maine.edu; 6 Centers for Disease Control and Prevention, National Center on Birth Defects and Developmental Disabilities, Atlanta, GA 30333, USA; MGaffney@cdc.org; 7 Speech Pathology/Audiology, Veterans Health Care System of the Ozarks, Fayetteville, AR 72703, USA; winnie.chung@va.gov

**Keywords:** early intervention, deafness, frequency of intervention, intervention dosage, language outcomes, expressive vocabulary, predictors of outcomes

## Abstract

Background: A primary goal of early intervention is to assist children in achieving age-appropriate language skills. The amount of intervention a child receives is ideally based on his or her individual needs, yet it is unclear if language ability impacts amount of intervention and/or if an increased frequency of intervention sessions results in better outcomes. The purpose of this study was to determine the relationship between the frequency of early intervention sessions and vocabulary outcomes in young children with hearing loss. Methods: This was a longitudinal study of 210 children 9 to 36 months of age with bilateral hearing loss living in 12 different states. Expressive vocabulary skills were evaluated using the MacArthur–Bates Communicative Development Inventories. Results: A higher number of intervention sessions reported at the first assessment predicted better vocabulary scores at the second assessment, and more sessions reported at the second assessment predicted better scores at the third assessment. For each increase in the number of sessions reported, there was a corresponding, positive increase in vocabulary quotient. In contrast, children’s vocabulary ability at an earlier time point did not predict intervention session frequency at a later point in time. Conclusions: A significant prospective effect was apparent with more therapy sessions resulting in improved vocabulary scores 9 months later. These findings underscore the importance of early intervention. Pediatricians and other health care professionals can help apply these findings by counseling parents regarding the value of frequent and consistent participation in early intervention.

## 1. Introduction

Early Hearing Detection and Intervention (EHDI) programs have been established to help ensure all infants receive recommended hearing screening, diagnostic, and early intervention services. EHDI programs follow the Joint Committee on Infant Hearing (JCIH) 1-3-6 guidelines, which state that all newborns should be screened for hearing loss by one month of age, and those with hearing loss should be identified by 3 months and enrolled in intervention by 6 months of age. Children who are deaf or hard of hearing who meet these guidelines, on average, achieve higher language skills than those who do not [1]; however, as a group, children with hearing loss often still do not achieve age-appropriate language milestones [1,2,3,4,5].

### 1.1. Amount of Intervention and Developmental Outcomes

The JCIH recommends that children with hearing loss receive early intervention with the goal of promoting development of age-expected speech and language outcomes [6]; however, there is not a recommended prescribed dosage of therapy. The question of “how much intervention is enough to achieve desired outcomes?” is relevant to policy makers and practitioners alike. Too much intervention may use limited resources and burden the child and caregiver(s) unnecessarily, while too little intervention may result in lost opportunity to promote language milestones at a prime developmental period.

In contrast to fields such as pharmacology in which dosage and frequency of specific medications are well documented [7], relatively little is known about optimal frequency and intensity of intervention based on structured developmental and behavioral therapy [8,9]. Interventions targeting language development are difficult to evaluate due to nuances such as language levels influencing the number of sessions a child receives. While there is evidence to indicate that intensive therapy is required in order to influence the neurophysiological basis of various impairments [10], little is known about optimal intervention intensities in speech-language pathology services among young children who are deaf or hard of hearing [8]. This type of intervention targets the speech, language, and listening skills of the child as well as facilitating language strategies used by the family. The limited amount of existing evidence on the impact of early intervention shows that more service leads to better outcomes [11,12,13].

### 1.2. The Current Study

To help quantify intervention effectiveness, Warren and colleagues [9] proposed specific terms to describe the variables that make up intervention intensity including “dose frequency,” which they defined as the number of intervention sessions per unit of time. It is this aspect of intervention intensity that was examined in the current study. Specifically, the purpose of this study was to assess the relationship between the number of early intervention sessions per month and expressive vocabulary outcomes in young children with hearing loss.

## 2. Materials and Methods

### 2.1. Participants

This was a longitudinal study of 210 children with bilateral hearing loss. Participants ranged from 9 through 36 months of age. Based on parent and interventionist report, the children had no additional disabilities thought to impact language development. Participants lived in 12 states (Arizona, California, Florida, Idaho, Indiana, Maine, New Mexico, North Dakota, Texas, Utah, Wisconsin, and Wyoming) and were from homes where the primary language was English or American Sign Language. All children were participating in a multistate project, supported by the Centers for Disease Control and Prevention, examining developmental outcomes of young children with hearing loss. Demographic characteristics of the participants and their parents are summarized in Table 1.

#### 2.1.1. Characteristics of Participants’ Hearing Loss

Children’s degree of hearing loss ranged from mild to profound, with 60% of children having a documented mild or moderate hearing loss and 40% having a moderate–severe to profound loss. The onset of hearing loss was congenital for 97% of the participants. For the remaining 3%, hearing loss was acquired prior to 8 months of age. The majority of children (74%) met EHDI 1-3-6 guidelines and 90% used some form of amplification. By the third assessment, 24% of the children used a cochlear implant. For details about the children’s hearing loss, see Table 2.

#### 2.1.2. Frequency of Participants’ Intervention

Families received individual early intervention services in their homes and/or an intervention center. Early interventionists were from a variety of professional backgrounds including teachers of the deaf, speech/language pathologists, and early childhood specialists. Information on the mean number of individual sessions received per month (i.e., “the number of sessions per month your child/family typically receives”) was gathered from a demographic form completed at each assessment by either the interventionist or the family. 

### 2.2. Procedures

#### 2.2.1. Data Collection Instruments

All families and/or their interventionist completed a demographic form, which included information such as the caregivers’ level of education, age of hearing loss identification, and amount and type of intervention. Audiologic records were reviewed by study personnel to determine a child’s degree of hearing loss. At each assessment, the participants’ expressive vocabulary (signed and/or spoken) was measured based on caregiver report using the MacArthur–Bates Communicative Development Inventories (Mac:CDI, Brookes, Baltimore, MD, USA) [14]. This norm-referenced assessment has been validated with typically developing children [15,16] as well as those with hearing loss [17,18]. Expressive vocabulary was selected as the outcome variable because vocabulary size and rate of word learning are important predictors of later language and academic skills [19,20].

#### 2.2.2. Administration of Expressive Vocabulary Assessment

In keeping with the administration instructions for populations with language delays, the level of the inventory administered (Words and Gestures versus Words and Sentences) was determined by the interventionists’ and/or caregivers’ estimate of the child’s vocabulary size [14]. For participants in all but one state, the appropriate MacArthur inventory was given to the family by their early interventionist. In the remaining state, the inventory was mailed to the family’s home. The Mac:CDI lists a variety of early-developing words. The child’s primary caregiver was instructed to mark all words their child produced spontaneously in spoken and/or sign language. The form was then reviewed by the child’s early interventionist for completeness and accuracy and sent to the project staff for scoring.

#### 2.2.3. Scoring of Expressive Vocabulary Assessment

Assessment scoring was completed by one person and checked by a second person. Disagreements in scoring were resolved by consensus. Total raw scores were calculated by counting the number of words a child produced regardless of modality (spoken or signed). Raw scores were converted to vocabulary age scores using the procedure described in the test manual. To examine each participant’s expressive vocabulary age score relative to their chronological age, vocabulary quotients (VQs) were calculated by dividing the child’s vocabulary age by their chronological age and multiplying by 100. A VQ of 100 indicated a child’s vocabulary age was commensurate with his or her chronological age.

### 2.3. Data Analysis: Structural Equation Model

In order to test the causal direction between the frequency of intervention and expressive vocabulary, primary analyses used a three-wave, cross-lagged panel design structural equation model, with cross-lagged effects between the number of sessions reported and the child’s language score. Full information maximum likelihood estimation was used in order to address missing data. Maternal level of education, degree of hearing loss (moderate–severe to profound vs. mild/moderate), and EHDI 1-3-6 status were included as control variables, and all three were allowed to correlate. All three were tested as predictors of both Time 1 vocabulary and the number of sessions at Time 1. In addition, maternal level of education was evaluated as a predictor of the Time 2 and Time 3 language scores. Furthermore, in order to address anticipated ongoing impacts of hearing loss on language development and service needs, degree of hearing loss was allowed to predict language scores and service levels at all three time points. See Table 3 for a description of the coding of the independent variables included in the model.

Recognizing the developmental process of language skills, language scores at Time 1 predicted Time 2 language scores, with Time 2 language scores predicting Time 3 language scores. Similarly, anticipating some continuity of care, the number of sessions reported at Time 1 predicted the number of sessions at Time 2, and Time 2 sessions predicted Time 3 sessions. Residuals for language scores at all three time points were allowed to correlate. Similarly, residuals for the number of sessions at Time 1 and Time 2 were allowed to correlate, as were residuals at Time 2 and Time 3. Correlated residuals between sessions at Time 1 and Time 3 resulted in a Heywood case and were not included. Finally, the language and sessions residuals at each individual time point were allowed to correlate.

### 2.4. Research Questions

The core questions in this study were (1) whether a relationship exists between the frequency of services a child with hearing loss receives and expressive vocabulary scores and, (2) the causal direction of any such relationship. Given that there are three time points, there are two sets of cross-lags: Time 1 language score and therapy sessions predicting Time 2 values of the other; and Time 2 language score and therapy sessions predicting Time 3 values of the other.

## 3. Results

### 3.1. Preliminary Analyses: Skew and Kurtosis

To reduce the skew in the number of intervention sessions, participants were limited to those who reported no more than 10 sessions per month throughout the course of this study. While this improved both skew and kurtosis, a square root transformation was applied to further reduce this issue. This resulted in a skew of −143 (SE = 0.109) and kurtosis of −155 (SE = 0.192) in the transformed variable.

### 3.2. Descriptive Statistics

Data for this analysis were based on assessments that occurred at regularly spaced intervals for each child, with approximately 9 months elapsing between assessments. Three assessment periods were identified. The first (Time 1) was when the children were between 9 and 16 months of age (mean = 13.3; SD = 2.04). The second assessment (Time 2) occurred between 17 and 26 months of age (mean = 22.3 months; SD = 2.54), and the third assessment (Time 3) was between 27 and 36 months of age (mean = 31.5; SD = 2.33). All participants were assessed on at least two occasions with all 210 children receiving an assessment at Time 1, 164 assessed at Time 2, and 130 with an assessment at Time 3. Some children were unable to be assessed at all three time points depending on the date of their enrollment in this study and when this study concluded.

As shown in Table 4, Mac:CDI vocabulary quotients decreased over time, declining from 94.3 at the initial assessment to 73.7 at the third assessment (t(129) = −10.203, *p* < 0.001). The number of sessions per month increased from a mean of 3.4 at the first assessment to a mean of 4.0 per month at the third assessment (t(129) = 3.280, *p* = 0.001). Intervention sessions ranged from 30 through 90 minutes in length, with approximately 80% of the sessions being 60 minutes long.

### 3.3. Structural Equation Model Results

**Figure 1:**
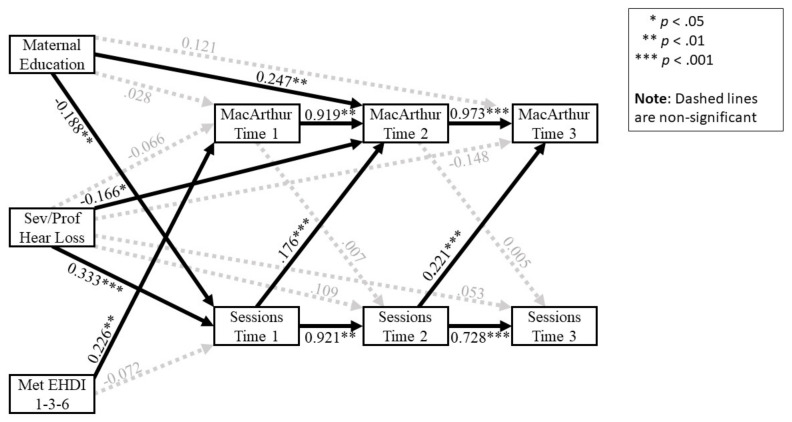
Language and intervention cross-lagged effects in children 9 to 36 months of age with bilateral hearing loss.

The resulting structural equation model with standardized coefficients is presented in Figure 1. Paths with *p* < 0.05 appear in solid black; non-significant paths appear in grey, hashed lines. Preliminary analyses suggested that the coefficients for each similar pair of cross-lagged effects (e.g., Time 1 sessions predicting Time 2 language, and Time 2 sessions predicting Time 3 language) could be constrained equal in order to produce a more parsimonious model (χ^2^(2, N = 210) = 3.105, *p* = 0.212). Fit indices suggested that the data fit the model well (χ^2^(7, N = 210) = 5.950, *p* = 0.546; CFI = 1.000; RMSEA = 0.000, CI_90%_ = [0.000, 0.077]).

#### 3.3.1. Relationship of Control Variables to Expressive Vocabulary Scores

Meeting EHDI 1-3-6 guidelines was associated with higher Time 1 vocabulary scores (β = 0.226, C.R. = 3.378, *p* < 0.001). Based on the unstandardized path coefficient (b = 10.251), this translated to a 10.25-point increase in Time 1 language quotients for children meeting the EHDI 1-3-6 guidelines. Having a moderate/severe to profound hearing loss, rather than a mild/moderate hearing loss, was associated with more sessions at Time 1 (β = 0.333, C.R. = 5.077, *p* < 0.001), but had no additional impact at Time 2 (β = 0.109, C.R. = 1.017, *p* = 0.309) or Time 3 (β = 0.053, C.R. = 0.699, *p* = 0.485). A moderate/severe to profound hearing loss, rather than mild/moderate, was also associated with lower vocabulary scores at Time 2 (β = −0.166, C.R. = 2.127, *p* = 0.033). Maternal education was negatively related to the number of sessions at Time 1 (β = −0.188, C.R. = −2.829, *p* = 0.005), with children of more educated mothers initially receiving fewer sessions. Finally, maternal education was positively related to vocabulary scores at Time 2 (β = 0.247, C.R. = 3.150, *p* = 0.002), reflecting higher language skills for children of more educated mothers.

#### 3.3.2. Relationship of Intervention Frequency and Expressive Vocabulary Scores

Regarding the cross-lagged effects, results showed a significant prospective effect of an increased number of sessions reported at Time 1 predicting future vocabulary scores at Time 2 (β = 0.176, C.R. = 3.956, *p* < 0.001), and for the number of sessions at Time 2 predicting future vocabulary scores at Time 3 (β = 0.221, C.R. = 3.956, *p* < 0.001). In contrast, language scores at Time 1 were not associated with the subsequent number of sessions at Time 2 (β = 0.007, C.R. = 0.135, *p* = 0.893), nor were language scores at Time 2 related to the number of sessions at Time 3 (β = 0.005, C.R. = 0.135, *p* = 0.893).

**Figure 2:**
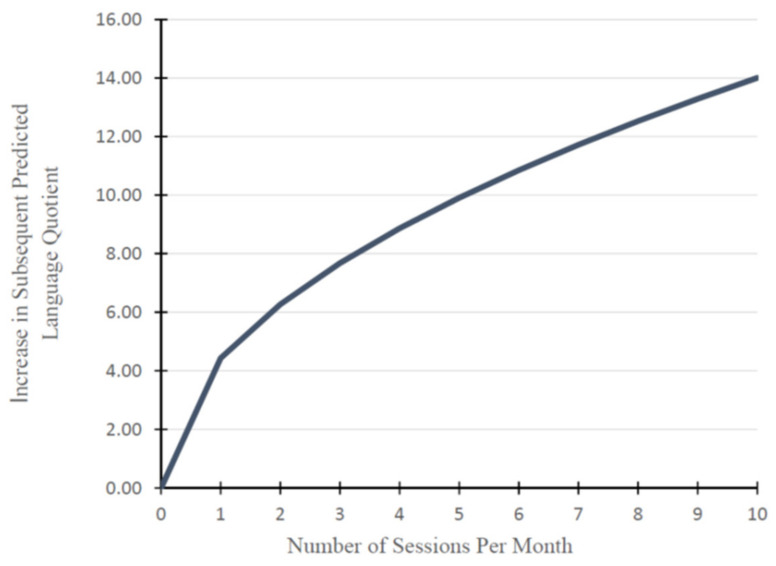
Increase in language quotient by the number of sessions reported per month.

To help interpret the prospective relationship between the number of sessions and language scores, Figure 2 shows the predicted impact of numbers of sessions (in their original units, not the square root transformed values used in the modeling) on subsequent language scores. For example, if a family reported receiving no sessions, there was no corresponding impact on a child’s language quotient score. In contrast, one session per month was associated with a 4.43-point increase in their predicted language quotient, while four sessions per month was associated with an 8.86-point increase, and 8 sessions per month was associated with a 12.53-point increase in their predicted language quotient.

## 4. Discussion

The present study investigated the impact of the number of intervention sessions per month on expressive vocabulary development in children 9 to 36 months of age with bilateral hearing loss across 12 states. In keeping with previous studies [11,12,13], increased frequency of early intervention sessions was associated with improved outcomes. Specifically, a greater number of sessions per month predicted higher vocabulary scores 9 months later when controlling for degree of hearing loss, maternal level of education, and meeting EHDI 1-3-6 guidelines.

### 4.1. Clinical Implications

It is important to establish the optimal dosage of intervention for children with hearing loss to help guide the delivery of effective services [8]. Additionally, it helps inform decisions by practitioners and policy makers about how to best allocate limited resources such as funds, therapist time, and family time. Given the findings that more sessions reported per month predicated higher expressive vocabulary scores, this study supports the adage that “more is better” for supporting vocabulary development among young children with bilateral hearing loss. While one intervention session per month resulted in a 4-point higher predicted vocabulary quotient than no sessions, this is insufficient for closing the significant gap that exists between vocabulary skills of children with hearing loss and their same-age peers.

On average, the families of children in this study reported receiving three to four intervention sessions per month, i.e., one session per week. While this is common practice across the United States, perhaps for fiscal reasons, it is clear from this work that more therapy service leads to improved outcomes. Specifically, the trajectory of predicted vocabulary scores based on the number of intervention sessions shown in Figure 2 indicates that children benefit from access to services beyond a once-a-week therapy schedule.

### 4.2. Relationship of Control Variables to Vocabulary Outcomes

In addition to a greater number of intervention sessions per month, in keeping with prior research, higher vocabulary scores were predicted by meeting EHDI 1-3-6 guidelines [1], higher levels of maternal education [1,21,22], and less severe degrees of hearing loss [1,5,23,24,25]. While maternal education and degree of loss predicted vocabulary scores at Time 2, the only significant predictor of vocabulary at Time 1 was meeting EHDI 1-3-6 guidelines. Vocabulary scores at Time 1 then predicted vocabulary at Time 2, which in turn predicted vocabulary at Time 3. This further highlights the importance of early identification and intervention for setting children on a trajectory of improved vocabulary outcomes throughout the birth to 3-year period.

### 4.3. Influence of Vocabulary Ability on Intervention Frequency

In contrast to the number of therapy sessions predicting later vocabulary outcomes, vocabulary ability did not predict the number of sessions the families received at subsequent points in time. Federal law requires that a child’s Individualized Family Service Plan (IFSP) includes the frequency of early intervention services provided to the family [26]. This decision should be based, in part, on the developmental needs of the child [26], i.e., the lower the expressive vocabulary, the greater service the family/child should receive. However, we see from this study that children’s vocabulary scores at an earlier assessment did not predict the amount of sessions reported at later time points. There are a number of possible explanations for this finding. It is possible that IFSP teams are focusing on other domains, such as speech intelligibility, rather than language skills, when determining the intensity of intervention services. It is also possible that programs prescribe a standard number of sessions per week/month regardless of degree of need and that this allocation does not change despite indications of a need for increased services [27].

### 4.4. Relationship of Control Variables to Intervention Frequency

In examining factors that influenced the number of intervention sessions families indicated they received, children with more significant degrees of hearing loss and those whose mothers had lower levels of education were more likely to report receiving a greater number of intervention sessions per month at Time 1. This practice is encouraging in that it is responsive to research establishing that children from families with lower maternal education and those with more significant hearing loss are at greater risk for language delay [1,5,21,22,23,24,25,26]. The consistency of this finding across multiple studies (including the current one) points to the importance of continuing to provide increased intervention for children exhibiting one or both of these characteristics.

### 4.5. Study Limitations and Directions for Future Research

A limitation of the current investigation is that it did not include children with additional disabilities nor a significant number of children using sign language, and so it does not represent the full population of children with hearing loss. Additionally, it relied on parent and interventionist report of the number of intervention sessions received per month. Although we had no reason to distrust the veracity of these reports, ideally the number of sessions over the course of the birth to 3-year period would be tracked objectively, such as via interventionists’ billing statements or visit logs. The potential for inaccuracies in the reported data was reduced by (1) obtaining reports of the number of sessions received rather than relying on the number prescribed in the IFSP, (2) requesting updates at 6-month intervals, and (3) receiving responses that were corroborated by both the family and interventionist. Future research may be able to provide greater detail on the specific topics of each therapy session and whether this has a differential impact on the outcomes of different developmental domains. Additionally, pairing quantity of early intervention with data from the same children on their long-term academic performance, vocational options, and mental health outcomes would help quantify the long-term cost–benefit of quantity of early intervention services.

## 5. Conclusions

In this study, the number of intervention sessions per month predicted later vocabulary scores, with more intervention leading to improved expressive vocabulary outcomes during the birth to 3-year period for children with bilateral hearing loss. This underscores the importance of intervention in the outcomes of children who are deaf or hard of hearing. These findings also highlight the important role that pediatricians and other health care providers such as audiologists, otolaryngologists, and speech-language pathologists can serve in encouraging families of children with hearing loss to enroll in intervention services that meet their needs, attend sessions consistently, and advocate for increased services. Parents often look to their child’s primary care providers for advice, and these professionals can help families understand the important relationship between high participation in intervention services and positive vocabulary outcomes, which, in turn, is associated with positive impacts such as learning to read and development of higher-level language skills.

## Figures and Tables

**Table 1:** Participant and Family Demographic Characteristics.

Characteristic	Percentage of Participants
Sex	
Male	47%
Female	53%
Ethnicity	
Non-Hispanic	80%
Hispanic	20%
Race	
White	87%
African American/Black	2%
Asian	2%
Native American	1%
Hawaiian/Pacific Islander	1%
Mixed race	7%
Communication mode used with the child	
Primarily spoken language	79%
Spoken language only	28%
Spoken with very occasional use of sign	51%
Sign language + spoken language	19%
Sign only	2%
Hearing status of the parent	
Both parents hearing	81%
One or both parents deaf/hard of hearing ^a^	19%
Mother’s highest educational degree	
Less than high school	7%
High school	36%
Vocational	5%
Associate’s	15%
Bachelor’s	26%
Graduate	11%

^a^ Of the parents who were deaf or hard of hearing, 55% used sign language when communicating with their child.

**Table 2:** Characteristics of the Participants’ Hearing Loss.

Characteristic	Percentage of Participants
Degree of hearing loss	
Mild to moderate	60%
Mild (26 to 40 dB HL)	37%
Moderate (41 to 55 dB HL)	23%
Moderate–severe to profound	40%
Moderate–severe (56 to 70 dB HL)	11%
Severe (71 to 90 dB HL)	9%
Profound (>90 dB HL)	20%
Onset of Hearing Loss	
Congenital	97%
Acquired prior to 8 months of age	3%
Met EHDI 1-3-6 guidelines	
Yes, met guidelines	74%
No, did not meet guidelines	26%
Type of amplification used	
None	10%
Hearing aids	76%
Cochlear implant	9%
Bone conduction hearing aid	3%
Hearing aid + cochlear implant	2%

Note. dB HL = decibels in Hearing Level. Degree of hearing loss was determined based on the better-ear pure tone average (PTA), i.e., the average of hearing thresholds at 500, 1000, and 2000 Hz. By the third assessment, 24% of the children used a cochlear implant.

**Table 3:** Description of the Coding of the Independent Variables Included in the Structural Equation Model.

Independent Variable	Coding of Variable
Adherence to the EHDI 1-3-6 guidelines	0 = does not meet 1-3-6 guidelines;
	1 = meets 1-3-6 guidelines
Maternal level of education	Continuous variable: 1 year increments
Degree of hearing loss	0 = mild/moderate levels;
	1 = moderate–severe to profound levels
Sessions	Continuous variable: number per month

Note. EHDI = Early Hearing Detection and Intervention; 1-3-6 guidelines = hearing screening by 1 month of age, identification of hearing loss by 3 months of age, and enrollment in intervention by 6 months of age.

**Table 4:** Means and Standard Deviations for Vocabulary Quotients and the Average Number of Intervention Sessions Per Month.

	Mean	SD	N
Mac:CDI vocabulary			
quotient			
Time 1	94.3	20.0	210
Time 2	82.0	14.9	164
Time 3	73.7	14.5	130
Number of sessions			
Time 1	3.4	2.1	210
Time 2	4.0	2.7	164
Time 3	4.0	2.8	130

Note. Mac:CDI = MacArthur–Bates Communicative Development Inventories.

## Data Availability

The data presented in this study are available on request from the corresponding author. The data are not publicly available because consent for this was not obtained from the participants.
